# Photobiomodulation by a 635nm Diode Laser on Peri-Implant Bone: Primary and Secondary Stability and Bone Density Analysis—A Randomized Clinical Trial

**DOI:** 10.1155/2019/2785302

**Published:** 2019-04-22

**Authors:** Jacek Matys, Katarzyna Świder, Kinga Grzech-Leśniak, Marzena Dominiak, Umberto Romeo

**Affiliations:** ^1^Private Dental Practice, Lipowa 18, Wschowa, Poland; ^2^“Sapienza” University of Rome, Italy; ^3^The Prosthodontic Specialist Training Program, Dental Prosthodontics Clinic, Medical University of Wroclaw, Poland; ^4^Dental Surgery Department, Medical University of Wroclaw, Poland; ^5^Department of Oral and Maxillofacial Sciences, “Sapienza” University of Rome, Italy

## Abstract

**Introduction:**

Various procedures in dental implantology are performed to enhance the bone healing process and implant stability. One of these methods can be a low-level laser therapy (LLLT).

**Objectives:**

The aim of our study was to evaluate the stabilization (primary and secondary) and bone density in peri-implant zone after LLLT protocol using a 635 nm diode laser.

**Material and Methods:**

The research included 40 implants placed in the posterior region of a mandible in 24 patients (8 women and 16 man; age: 46.7 ± 8.7 years). The patients were randomly divided into 2 groups G1 (n=12, 18 implants) and G2 (n=12, 22 implants) according to the treatment procedure; G1 (test): 635 nm laser, with handpiece diameter: 8mm, output power: 100mW, spot area: 0.5024cm^2^, average power density: 199.04mW/cm^2^, continuous mode, dose: 4J per point (8J/cm^2^), time: 40 sec per point, 2 points (irradiation on a buccal and a lingual side of the alveolus/implant), and total energy per session 8J; G2 (control): no laser irradiation. The G1 (test) group's implants were irradiated according to the following protocol: 1 day before surgery, immediately after the surgery and 2, 4, 7, and 14 days after. The total energy after all therapeutic sessions was 48J. The implants stability was measured employing a Periotest device (Periotest Test Value: PTV) (measured immediately after the surgery, 7 days, 2 weeks, 4 weeks, and 2 and 3 months after the surgery) and the bone density using cone-beam computed tomography (grayscale value) (measured immediately after the surgery, 4 weeks and 12 weeks after the treatment).

**Results:**

The average implant stability at different time points showed lower PTV value (higher stability) at 2^nd^ and 4^th^ week after 635 nm laser irradiation (G1) compared with a control (G2) group (p<0.01). The secondary stability of the implants after 12 weeks observation was not significantly higher for the laser group in contrast to none-irradiated implants (p>0.05). The mean grayscale value at the apical, middle, and cervical level of the titanium implants showed the reduction of pixel grayscale value after 2 weeks and was lower for the G1 group in contrast to the G2 group (p<0.01). The value of grayscale after 12 weeks was significantly higher at the middle and apical level of the implants in the G1group in contrast to the G2 group (p<0.01).

**Conclusion:**

The application of the 635 nm diode laser enhanced secondary implant stability and bone density. However, to assess the impact of the LLLT on peri-implant bone with different bone densities, further well-controlled long-term trials on larger study groups are needed.

## 1. Introduction

Dental implants have been used for missing teeth replacement with a high success rate [1, 2]. Bone quantity and quality as well as osseointegration process are one of the most important factors responsible for the long-term clinical success in their implantation [3]. Additionally, adequate primary stability of implants is a key factor enabling their immediate or early loading [4]. Implant stability is defined as a biomechanical stability upon implant insertion and depends on bone formation at the bone-implant interface [5, 6]. The degree of achieved primary stability includes bone quality and quantity, implant morphology, implant surface characteristics, and surgical technique [7].

One of innovative methods to enhance the process of bone healing and at the same time increasing the primary stability is a low-level laser therapy (LLLT) [8]. The LLLT involves the application of a monochromatic light with a low energy density which induces nonthermal photochemistry effects on cellular level [9]. Several studies documented an increase in the stability of implants and the bone-implant contact (BIC) factor, after implant laser irradiation [10–12]. LLLT laser with low-energy density range stimulates the mitochondial and cellular membrane photoreceptors to synthesize ATP, which enhances cell proliferation rate [13, 14]. The laser also has a biostimulatory effect on bone tissue by increasing proliferation and differentiation of osteoblasts [15–17]. Additionally, the research by AlGhamdi et al. [13] revealed that LLLT can induce mitosis in cultured cells, collagen production, and DNA and RNA synthesis. Several studies showed that the use of lasers in soft and hard tissue surgeries improves and accelerates healing [16, 18–23]. Moreover, Mohammed et al. [24] in his study demonstrated that the LLLT reinforces the revitalization process, enhances the healing of injured tissues, and promotes nerve regeneration.

Furthermore, the adequate method of measuring effectiveness of primary stability and bone density is required. Since the removal torque method and histomorphometric analysis measurements are invasive techniques [23], Periotest and resonance frequency analysis (RFA) are more often used to assess the implant stability [5, 25–27]. The range of Periotest values (PTV) depends on the damping characteristics of the surrounding periodontium and its analysis proves to be of a clinical interest [25–27]. Additionally, cone-beam computed tomography (CBCT) has been reported to provide submillimeter isotropic voxels which allows accurate bone density measurements (error<0.1 mm) [28]. The method can be considered a preferential diagnostic tool for the bone density evaluation during implant treatment as it provides qualitatively and quantitatively analysis [25, 29].

There are only few studies that assess the LLLT effects on primary stability of implants; however they do not measure the possible bone density change [8, 30]. The aim of this study was to evaluate, by means of Periotest and CBCT, the effects of a 635 mm diode laser on implant stability, as well as bone density.

## 2. Material and Methods

The trial was designed as a randomized and controlled test. The approval of the Local Ethics Committee of Wrocław Medical University, Faculty of Dentistry, was obtained (permission numer: KB - 545/2018) and informed consent in accordance with the Helsinki Declaration was obtained from all participating subjects.

### 2.1. Subjects

**Figure 1:**
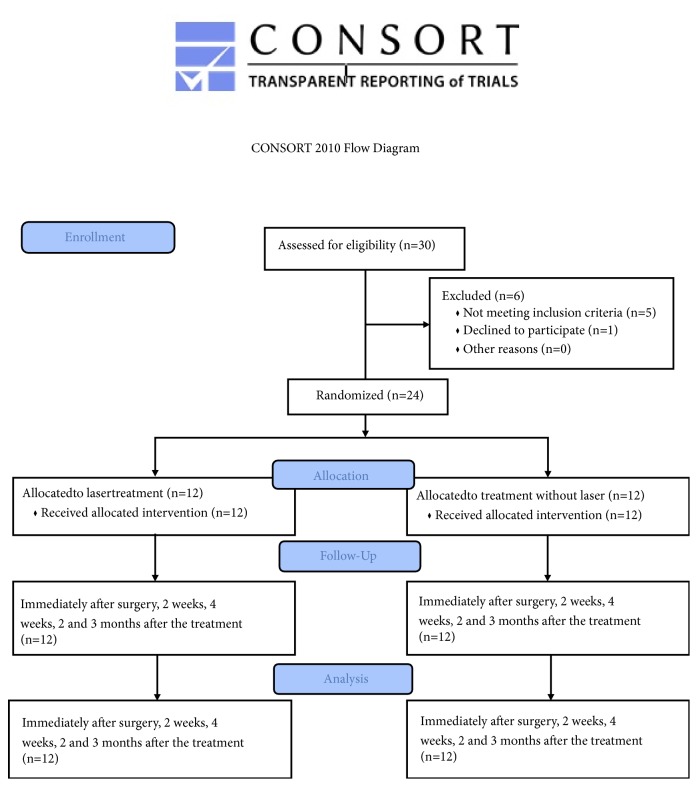
Flowchart of treated subjects according to CONSORT2010.

The study involved an insertion of 40 implants in total, in the posterior region of a mandible in 24 patients (8 women and 16 man; age: 46.7 ± 8.7 years) (Figure 1). All the patients were treated in the Private Dental Healthcare, Wschowa, Poland, by the same implantologist. The subjects were chosen for the study under the following inclusion criteria: partial edentulism in the left or right mandibular regions; no systemic diseases; were not using anti-inflammatory drugs; had not used antibiotics in the previous 24 months; were nonsmokers; had no uncompensated diabetes or uncontrolled periodontal disease; with bone density D2 (Misch's Classification)[31]; with bone division A[32]; no history of radiotherapy, or taking bisphosphonate medication; each patient has undergone hygienist treatment before the clinical trial.

### 2.2. Surgical Procedures

A total number of 40 implants (Superline, Daegu, Korea), made of titanium alloy (grade 4), 10-12 mm long with a diameter of 4.5 mm, were placed in the posterior region of the mandible. A total of 24 patients were randomly assigned with the use of a computer program to the 2 groups according to the treatment procedure: G1 (n=12, 18 implants) and G2 (n=12, 22 implants). In the posterior area of the mandible, a full- thickness mucoperiosteal flap has been elevated using one horizontal cut and 15C scalpel blade. The flap was detached, and an implant bed with the width of 4.5 mm was prepared using drills according to a manufacturer's protocol. The healing abutment was placed, and then the flap was sutured. After the procedure, additional antibiotic treatment was prescribed: Clindamycin (Clindamycin-MIP®, MIP Pharma, Innsbruck, Austria) in dose of 600mg, two times a day for 1 week, Ibuprofen (Ibuprom Max, US Pharmacia, Poland) in dose of 400mg, two times a day for 2 days, and 10 ml of 0.1% chlorhexidine mouthrinse (Eludril, Pierre Fabre, France) for 60 seconds, 3 times a day for 2 weeks.

### 2.3. Laser Application

**Figure 2:**
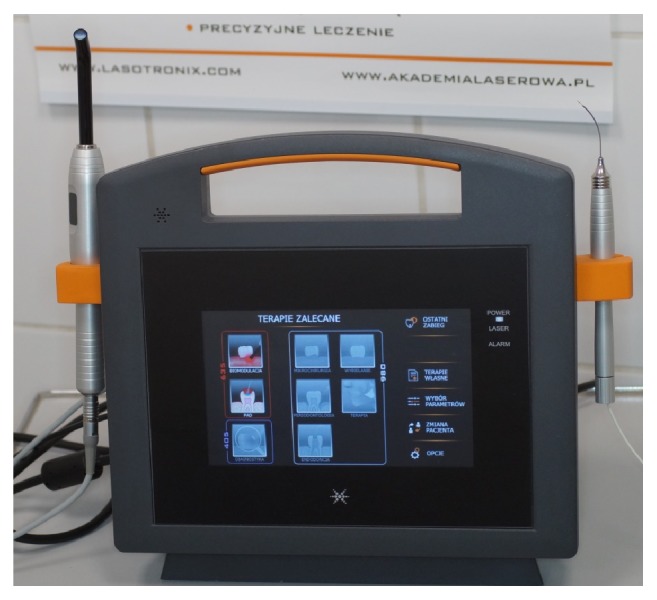
635 nm diode laser used in the study (SmartM, Lasotronix, Poland).

In our study we applied a red diode laser (SmartM, Lasotronix, Poland) at 635 nm wavelength with biomodulating handpiece with following set parameters: output power: 100mW, handpiece diameter: 8mm, spot area: 0.5024cm^2^, average power density: 199.04mW/cm^2^, continuous mode, dose: 4J per point (8J/cm^2^), time: 40 sec per point, 2 points (irradiation on a buccal and a lingual side of the alveolus/implant), and total energy per session 8 J. The diode laser was used in contact mode with peri-implant soft tissue only for the G1 (test) group according to the following irradiation protocol: 1 day before surgery, immediately after surgery and 2, 4, 7, and 14 days after. The total dose after all therapeutic sessions was 48J (Figure 2).

### 2.4. Measurement of Implants' Stability

**Figure 3:**
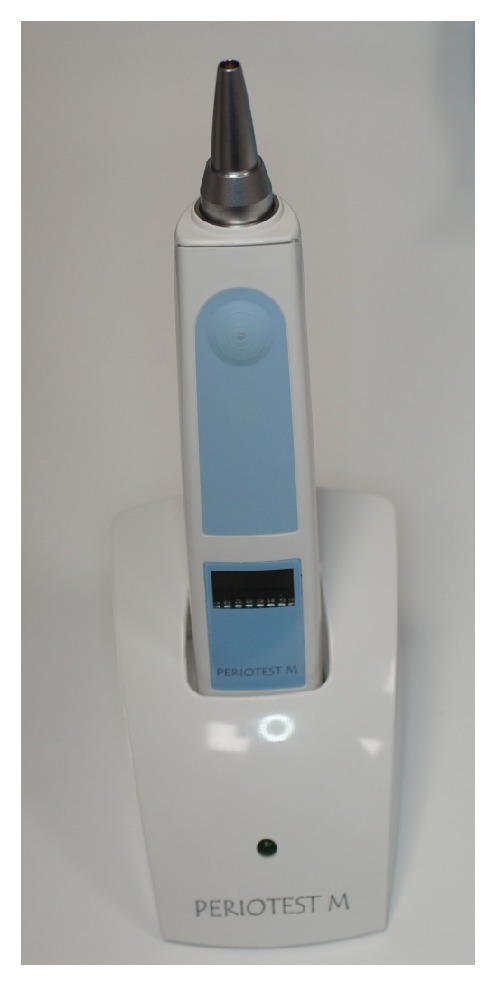
Periotest device used for implant stability measurement.

The implants stability was measured employing a Periotest device (Medzintechnik Gulden e K, Modautal, Germany). The Periotest measurement method includes the sound formed from contact between an object and a metallic tapping bar in a handpiece, which is electromagnetically pushed and electronically controlled. The Periotest response detection is analyzed through a fast Fourier transform (FFT) algorithm. Simply put, Periotest answer to tapping is estimated by an accelerometer and then analyzed. The signal produced by tapping is then transformed to a value called the Periotest value (PTV), which depends on the damping properties of peri-implant tissue. [25] The Periotest Test values (PTVs) are based on a numerical scale ranging from −8 to +50, determined by a mathematical computation. The lower Periotest values indicate higher implant stability and thereupon the higher absorption effect of the target tissues. Measurement of implant stability in the study was conducted: immediately after the surgery and 2 weeks, 4 weeks, and 2 and 3 months after the treatment. In each follow-up period, the measurements were done 5 times and mean results were assessed and compared (Figure 3).

### 2.5. Measurement of Bone Density

**Figure 4:**
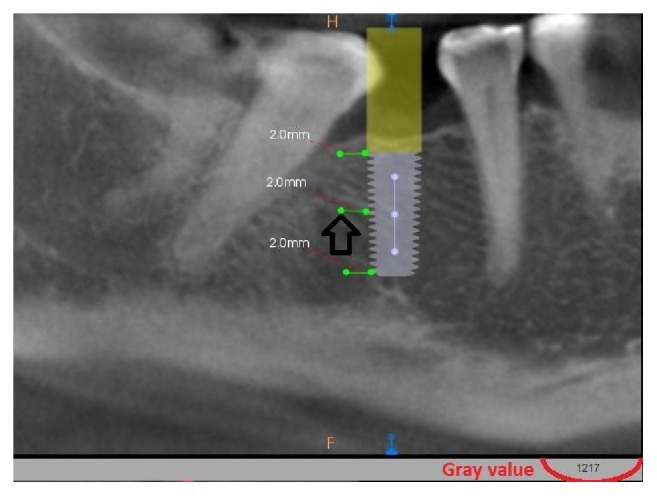
Calculation of grey value in CBCT. Dark arrow indicates the value of grayscale measured in the area of arrowhead (results highlighted in red color).

The surgery area and position of the implant after insertion were verified by cone-beam computed tomography (CBCT) examination (Kodak 9000 3D, Carestream/Trophy, Marne-la-Vallée, France), with a Field of View (FOV) equal 5x4 cm, nominal beam of 73 kV, 12 mA, and a voxel size of 90 *μ*m. The bone density was valued using a software Carestream 3D Suite (Carestream Health, Inc., Rochester, USA). The bone density (grayscale value) was measured at the three levels; cervical, middle, and apical part of each implant. The greyscale value for all subjects was measured by CBCT software at a distance of 2 mm from the implant to avoid the influence of the titanium artifact within 0.5mm perimeter. Measurement of the bone density was performed: immediately after the surgery, 4 weeks, and 3 months after the treatment (Figure 4).

### 2.6. Statistical Analysis

To assess whether the data were normally distributed, the Kolmogorov-Smirnov test was performed at the 95% level. The statistical analysis was performed utilizing repeated ANOVA measures and Bonferroni test to compare the mean implant stability values in the test and control groups over time. Differences in pixel grayscale values at the distal cervical, middle, and apical level of each implant of the two independent groups were compared with the Student t-test with the use of the program Statistica 12 (StatSoft, Krakow, Poland) at a significance level of p=0.05.

## 3. Results

**Figure 5:**
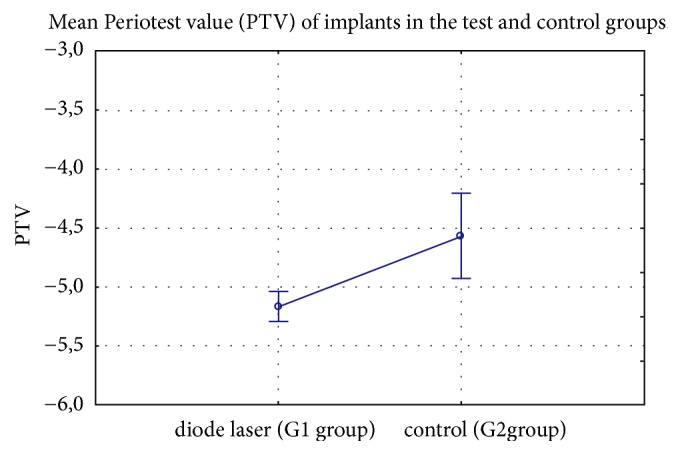
Mean Periotest value (PTV) of implants in the test and control groups.

The lowest mean PTV (-5.17±0.57) for inserted implants was measured in laser group (G1) compared to control group (-4.57±1.42) (p= 0.0026) (Figure 5).

The analysis of the implants' primary stability conjugated with PTV revealed significantly higher primary stability (lower PTV) for subjects irradiated with a 635 nm diode laser in contrast to nonirradiated patients after two (p<0.01) and four (p<0.05) weeks. (Table 1)

**Figure 6:**
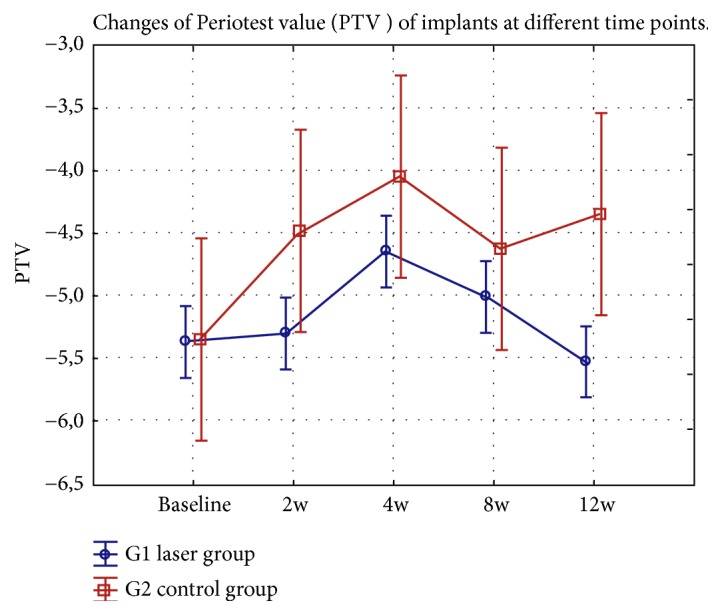
Changes of Periotest value (PTV) of implants at different time points (weeks: w) in the test and control groups.

The results showed that the implants' stability in both groups falls until the 4th week and then starts to increase again. In the laser group, the decrease in the stability was only minimal after 2 weeks in contrast to control subjects. Moreover, the secondary stability after 3 months measured in laser group was higher than at baseline, unlike control group where the secondary stability decreased (Figure 6).

Results of correlation of the average implant stability at different time points in the laser (G1) and control (G2) groups showed lower PTV value (higher stability) after 635 nm laser irradiation between the baseline and the 2nd, 4th, and 8th week (p<0.01). The secondary stability of the implants after 12 weeks observation was higher for the laser group in contrast to nonirradiated implants; however the differences were not significant (p=0.2759) (Table 2).

The mean grayscale value at the three levels, apical, middle, and cervical of titanium implants with or without 635 nm diode laser irradiation, was assessed. At all levels, the reduction of pixel grayscale value after 4 weeks was found to be significant for both the laser and the control group (p<0.01). The value of grayscale after 12 weeks was significantly higher at the middle and apical level of implants in the laser group in contrast to the control (p<0.01) (Tables 3–5).

## 4. Discussion

The LLLT is a noninvasive modality that can be reinforced to accelerate cellular processes such as synthesization of ATP [13, 14] and synthesis of DNA and RNA.[14] Many studies also proved its relevance in proliferation and differentiation of osteoblasts, bone healing and revitalization [8, 15–19], induction mitosis in cultured cells, collagen production[13], or even nerve regeneration [24]. Our study aimed at testing the photomodulation effect of the LLLT on implant stability and bone density after peri-implant soft tissue irradiation with a 635-mm diode laser by means of Periotest and CBCT analysis. The main finding of the study was that the subjects irradiated with a 635 nm diode laser accounted for significantly greater secondary stability (after 4 weeks) and bone density (after 12 weeks) in contrast to nonirradiated subjects. Similar value of primary stability was recorded in both subjects' groups (Table 1), which agrees with findings of other authors [11, 18, 30].

Low-level laser therapy in a range of 600-1100nm (optical window) results in a deeper tissue penetration and therefore evokes a broader cell-light response [6]. Arndt-Schultz's curve describes the dose-dependent effects of LLLT. It suggests that a low stimulus increases physiologic activity, moderate stimuli inhibit the activity, and very strong stimuli eliminate the activity [14]. That means that the use of insufficient dose has no biological effect but if too much energy is applied a biosuppressive effect will occur. The utilization of fluence in the range of 1–10 J/cm2 is optimal to receive an optimal biological response [6]. In our present study, a dose per point of 4J (8J/cm^2^) allowed increasing the secondary implant stability.

Moreover, we observed that the trend of reduction in implant stability was slower in the laser group in the first weeks and increased from the 6th to the 12th week as compared to the control group. The process of decrease and then increase in implant stability complies with findings of other studies [8, 30]. Significantly lower PTV (higher primary stability) was recorded in our research after two and four weeks for subjects irradiated with a 635 nm diode laser compared to the control group. In the laser group, the decrease in the stability was only minimal after 2 weeks. The implants' stability in both subjects' groups decreased until the 4th week but then started to increase again. The results may reflect the typical decrease of stability in bone healing process, followed by a rise or plateau in the subsequent weeks, as described by other investigators [30]. The secondary stability after 3 months was higher than at the baseline measured in the laser group, whereas in the control group is was lower, which complies with Gomes at al.[18] results at dose of 20J/cm^2^(830nm, 50mW). Nonetheless, the results of Torkzaban et al. [8] research were opted as of no clinical significance at dose of 4J – 14,18J/cm^2^ (100mW, 940nm). The different conclusions between the studies may result from differences in time of laser exposure, number of irradiations, periods between each treatment, and individual variability of each study group [33]. Moreover, Torkzaban et al. [8] used in their study the dose per square centimeter higher than was described as the optimal dose (1–10 J/cm^2^) by Arndt-Schultz's curve.

The second aim of our study was to determine the effect of the LLLT on bone density adjacent to dental implants using CBCT and mean grayscale values. Although some studies analyzed changes of BIC (bone to implant contact) after the LLLT [10, 18] none till now researched its effect on the bone quality and quantity. In our research lower bone density loss was noted at apical, middle implants' levels (lower reduction of pixel grayscale value) after 12 weeks for the laser group in contrast to control subjects. These findings may indicate the efficiency of the LLLT with a 635 nm diode laser in increasing secondary implant stability and bone density. In this regard, the results of our research could confirm the outcomes obtained from other studies such as improved BIC (bone-to-implant contact), implant stability, enhanced peri-implant bone repair, and bone neoformation [10, 11, 18].

The key to a clinical success in dental implantology is optimal osseointegration and implant stability [3–6]. In the present study, we found advantages of irradiating peri-implant soft tissue using a 635 nm diode laser which related to enhancing secondary implant stability and bone density. In the red to the near-infrared spectrum (600–1500nm), light scattering prevails, and absorption has less influence; thus the light penetrates to a depth of 8–10 mm [23, 34]. The penetration depth of a red laser is lower compared to the infrared one [35]. However, for the wavelength used in the study (635nm) the minimum penetration depth is around 3mm [34]. Application of the red laser at both lingual and buccal side of the mandible increases the total penetration depth; thus the energy can be absorbed by the soft tissue and bone. Therefore, because of the lower penetration depth of the red laser we recommend using the energy close to the maximal dose indicated by Arndt-Schultz's curve but less than 10J/cm^2^.

Taken together to ascertain a long-term clinical success in proposed method, additional trails using LLLT are required to confirm the results of our study to evaluate clinical application in implantation procedures. To assess the impact of the LLLT (red and infrared wavelengths) on peri-implant soft tissue, further randomized-controlled trials in long-term and larger study groups in contrast are warranted.

## 5. Conclusion

Irradiation of peri-implant soft tissue using a 635 nm diode laser enhanced secondary implant stability after four weeks and increased the grayscale value (bone density) after 12 weeks at the middle and apical level of the implant.

## Figures and Tables

**Table 1:** Results of Periotest value (PTV ) of implants at different time points in the test and control groups.

Period	Laser	Std	Control	Std	t-value	df	p-value
Basaline	-5.37	0.52	-5.35	0.68	-0.07	22	0.9468
2 weeks	-5.30	0.46	-4.48	0.53	-4.03	22	0.0006
4 weeks	-4.64	0.51	-4.04	0.59	-2.67	22	0.0141
8 weeks	-5.01	0.41	-4.63	0.59	-1.85	22	0.0779
12 weeks	-5.53	0.55	-4.35	2.89	-1.39	22	0.1797

**Table 2:** Mean differences in Periotest value (PTV ) of implants at different time points in the test and control groups.

Period	Mean differencess	CI		p value
		-95%	+95%	
Baseline vs 2 weeks	-0.88	-1.07	-0.70	0.0000
Baseline vs 4 weeks	-1.33	-1.61	-1.04	0.0000
Baseline vs 8 weeks	-0.74	-1.01	-0.47	0.0001
Baseline vs 12 weeks	-1.02	-2.97	0.93	0.2759
2 vs 4 weeks	-1.26	-1.57	-0.95	0.0000
2 vs 8 weeks	-0.68	-0.92	-0.43	0.0001
2 vs 12 weeks	-0.95	-2.89	0.99	0.3047
4 vs 8 weeks	-0.02	-0.27	0.24	0.8871
4 vs 12 weeks	-0.29	-2.27	1.68	0.7514
8 vs 12 weeks	-0.66	-2.65	1.33	0.4813

**Table 3:** Mean grayscale values of 2 groups at cervical level.

Period	Laser	Std	Control	Std	t-value	df	p-value
Baseline	1240.75	78.97	1283.42	66.66	-1.43	22	0.1667
4 weeks	755.92	34.51	726.83	24.18	2.39	22	0.0258
12 weeks	801.75	27.47	783.25	34.92	1.44	22	0.1633

Std: standard deviation.

df: degree of freedom.

**Table 4:** Mean grayscale values of 2 groups at middle level.

Period	Laser	Std	Control	Std	t-value	df	p-value
Basaline	1209.25	61.37	828.42	26.88	19.69	22	0.0000
4 weeks	573.42	20.96	551.17	34.35	1.92	22	0.0685
12 weeks	925.25	39.87	650.75	29.88	19.09	22	0.0000

Std: standard deviation.

df: degree of freedom.

**Table 5:** Mean grayscale values of 2 groups at apical level.

Period	Laser	Std	Control	Std	t-value	df	p-value
Baseline	1082.33	133.03	930.58	35.68	3.82	22	0.0009
4 weeks	465.83	34.80	700.33	47.34	-13.83	22	0.0000
12 weeks	708.08	121.43	439.50	27.06	7.48	22	0.0000

Std: standard deviation.

df: degree of freedom.

## Data Availability

The authors declare that they are in possession of complete data on the basis of which the results presented in the manuscript have been developed. The authors will make the data available to interested parties if necessary.
